# Comparison of severity of periodontal clinical parameters among naswar/snuff users: Cross sectional study

**DOI:** 10.1371/journal.pone.0273288

**Published:** 2022-09-16

**Authors:** Abid Rahim, Kawish Syed, Babar Ahad, Afaq Farooq, Zain Ayub, Syed Midhat Batool

**Affiliations:** 1 Department of Periodontology, Sardar Begum Dental College and Hospital (Gandhara University), Peshawar, Pakistan; 2 Department of Community Dentistry, Sardar Begum Dental College and Hospital (Gandhara University), Peshawar, Pakistan; 3 Department of Periodontology, CIMS Dental College, Multan, Pakistan; Federal University of Santa Maria: Universidade Federal de Santa Maria, BRAZIL

## Abstract

**Background:**

Naswar is a moist, non-chewable, and smokeless tobacco product ensconced in the buccal vestibule or floor of the mouth. Consumption of naswar is very popular in South Asia, especially Pakistan. This cross-sectional study compared the periodontal clinical parameters amongst mild, moderate, and severe naswar users.

**Methods:**

318 naswar users, categorized into three equal groups (n = 106) severe naswar users, moderate naswar users, and mild naswar users were drawn for this study. Bleeding on probing, pocking depth, gingival recession, and attachment loss were assessed using the UNC-15 probe and compared among the three groups of naswar users. Data analysis was done with the help of SPSS. Statistical significance was deliberated at *p* value ≤0.05.

**Results:**

28.9 ± 7.6 years was the mean age presentation. The mean % of bleeding on probing score was 61.95% in severe naswar users, 56.62% in moderate naswar users, and 51.23% in mild naswar users with a p-value of 0.001*. In severe, moderate, and mild naswar users the probing pocket depth (4-6mm) were 35.14%, 30.95%, and 23.21% respectively. 26.78% severe naswar users were having mean percentage for probing pocket depth (>6m) followed by moderate naswar users (17.26%) and mild naswar users (17.26%) with a significant p-value (0.001*). Clinical attachment loss (CAL) was 2.50 percent in light naswar users, 3.0 percent in moderate naswar users, and 4.25 percent in severe naswar users. Clinical attachment loss (CAL) was 2.50 percent in light naswar users, 3.0 percent in moderate naswar users, and 4.25 percent in severe naswar users. Severe naswar users had a high mean percentage of gingival recession (23.21%), whereas light 13.67 percent and moderate 14.88 percent naswar users had a smaller difference.

**Conclusions:**

Clinical periodontal parameters were more worsen in heavy naswar users compared to moderate and light naswar users.

## Introduction

Tobacco consumption is a grievous public health problem that kills more than eight million individuals a year globally [1]. According to the fact sheet of Global Tobacco Survey Pakistan (2014), about 19.1% (23.9 million) adults consumed tobacco in any variety. On the whole, 9.6 million adults (7.6%) use smokeless tobacco with men 11.4% and women 3.7%. At present time, the prevalence of smokeless tobacco consumption was higher in rural localities (8.2%) as compared to urban localities (6.7%) [2]. Smokeless tobacco (ST) is a large group of non-combusted tobacco products that are used orally or, much less often, breathed nasally, unlike smoked tobacco, which is burned or heated and then inhaled in items like cigarettes, cigars, pipes, or hookahs [3]. The usual and prevalent forms of smokeless and chewable tobacco in Pakistan include Naswar (moist snuff- ground leaves of dried tobacco), gutka (a blend of pulverized tobacco, betel nut, and hydrated lime), and pan (areca nut and crushed tobacco encased in betel leaf) [4,5].

**Fig 1:**
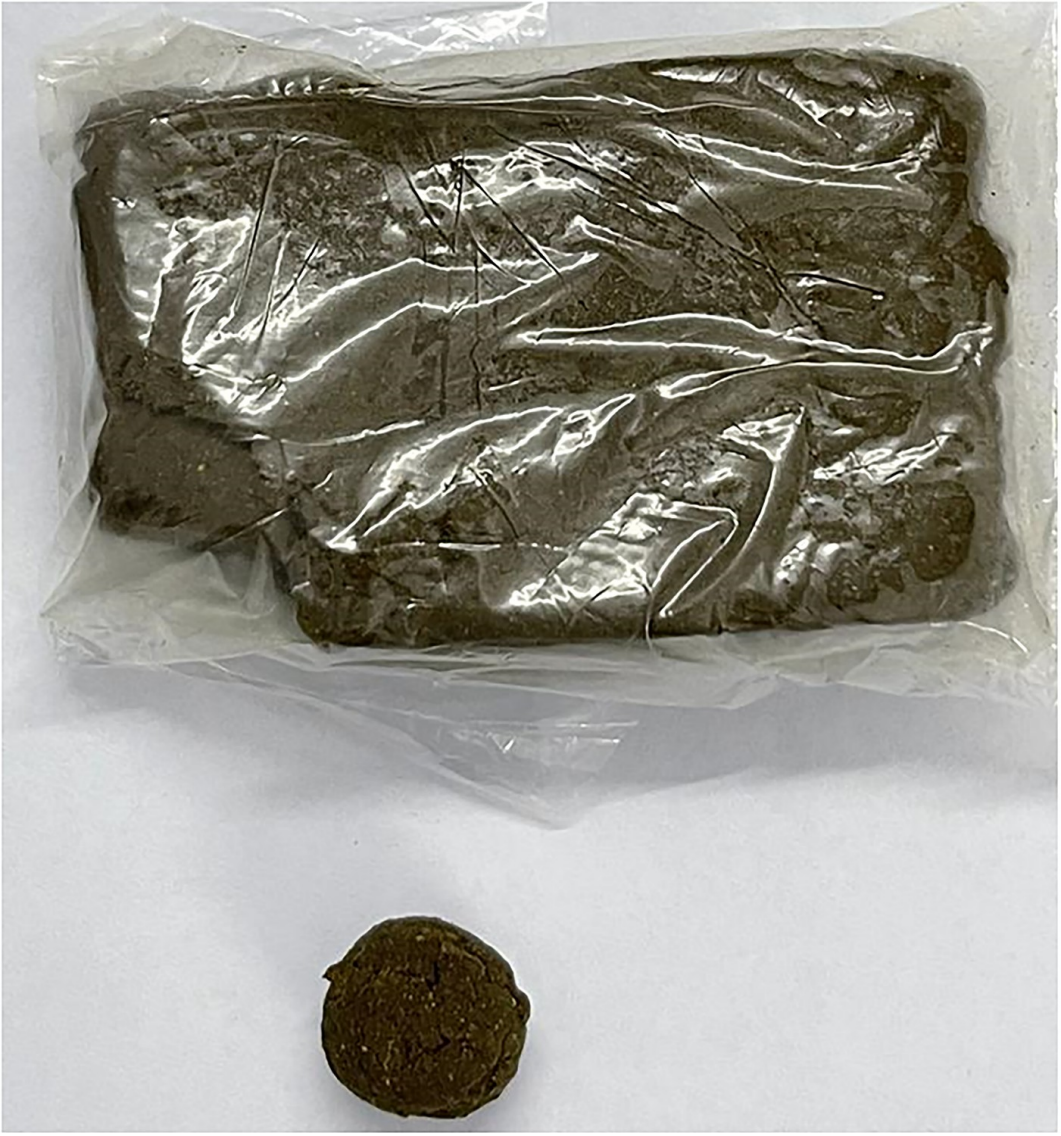
A packet of naswar with a glob.

Naswar is a moist, dipping, and non-chewable smokeless tobacco preparation made from an amalgamation of dried and pulverized tobacco leaves (*Nicotina rustica*), hydrated lime, ash of wood, and sometimes favorers like cardamom and menthol [4,6]. A small and desirable quantity of water is also added to this mixture to make it moist and pliable. Naswar has elevated pH and includes carcinogenic tobacco-related N-nitrosamines as well as some levels of cobalt, nickel, chromium, cadmium, arsenic, lead, and nitrate, which adversely affect oral health as well as general health [6,7]. More than 30 locally produced brands of naswar have pH between 8.5 to 9.0 with an average nicotine content of 14.6 mg/g. Naswar is routinely offered for sale in small plastic packets/bags (Fig 1). The average weight of each packet is approx. 45g [6,8]. Naswar is confined/ensconced in the labial/buccal vestibule or floor of the mouth as a glob for a certain time (15–30 minutes) before being expelled (without chewing), permitting the absorption of nicotine and other chemical substances through the oral mucosa [3,4,9]. Apart from other countries, it is most commonly used in Pakistan, Afghanistan, India, Iran, and Saudi Arabia [9].

The periodontium is harmed by tobacco use. This includes not just smoking but also smokeless tobacco [10]. Smokeless tobacco has previously been related to gingival recession and tooth loss. In comparison to non-tobacco users, tooth loss was shown to be significantly greater among smokers (56%) and smokers (58%) who used smokeless tobacco (28 percent) [11]. Smokeless tobacco (snuff- dry or wet) contains 28 carcinogens, and a prior study has connected the use of smokeless tobacco to a higher risk of cancer of oral squamous cells. Gingival recession and periodontal disease are the two most prevalent and possible side effects of using smokeless tobacco [12]. A study conducted by Khan et al. showed that Naswar users had a 20-fold higher oral cancer risk with OR 21.2 and 95% CI 8.4±53.5 when compared to non-users [8]. Habitual and prolonged consumption of smokeless tobacco wares (naswar) adversely affects the periodontium by local irritation and inflammatory process, causing worsening of pocket depth, bleeding upon probing, gingival recession, attachment loss, and, and loss of crestal bone [13–16]. According to Daood et al., the duration of naswar dipping had a substantial effect on the severeness of periodontal disease [15]. Smokeless tobacco users also develop lesions specified to the site (like submucosal fibrosis, keratosis, and lichenoid reactions) [17]. Snuff-induced lesions, on the other hand, appear to be reversible and vanish when snuff usage is stopped [7]. Many of the periodontal consequences of smokeless tobacco products might be attributed to local irritation and direct suppression of normal fibroblast activity as shown by the finding of Lallier et al. that smokeless tobacco extracts (STE) in greater concentrations (90μM) inhibited PDL and gingival fibroblast cell survival, post 24 hours [12]. There exist a dose-response relationship between smokeless tobacco use and severity of mucosal and periodontal injury as shown by the previous studies [12,18].

It is assumed by the hypothesis that the severity of periodontal clinical parameters increases with an increase in the dosage of naswar consumption. Immense use of Naswar by the local populace is solicitous and studies on the ramifications of Naswar’s dose on periodontal clinical parameters are scarce. Notably, the authors of this paper were unable to locate research that compared periodontal parameters in three groups: severe, moderate, and light Naswar users. The goal of this study was to assess and compare the severity of periodontal clinical parameters to the dose consumed. We will be able to properly counsel our patients on smokeless tobacco cessation if we have a better grasp of the dose-dependent effects of naswar on periodontal health.

## Materials and methods

### Ethical approval

This cross-sectional study was conducted in Sardar Begum Dental College and Hospital (Department of Periodontology) Peshawar, Pakistan from January 2019 to April 2019. The institutional Ethical Committee approved the study plan in its 17^th^ meeting. Written informed consent was acquired from all subjects who were volitional to participate and meet the requirements of the study.

### Inclusion criteria

i) Male patients (female patients were excluded due to cultural concerns and significantly less usage of naswar) with an age of 17–44 years. ii) Patients consuming naswar daily for≥12 months.

### Exclusion criteria

i) subjects who refused to participate in the study. ii) Current or former smokers using other forms of smokeless tobacco (gutkha, betel quid, pan). iii) Subjects who suffer from systemic conditions such as diabetes mellitus, cardiovascular diseases, etc. iv) Patients who have used antibiotics and steroids or anti-inflammatory medications for long term. v) Patients who presented with a history of periodontal treatment (non-surgical and surgical) in the last three months. vi) Patients with difficulty in mouth opening.

### Study participators and categorization

By using Open Epi software, the calculated sample size was 284 by taking probing pocket depth (PPD) 2.3 ± 0.3 in snuff users while 2.4±0.3 in controls, keeping 80% Power of the test and 95% confidence interval while keeping 20% non-responding rate, we took total sample size of around 318 participants [19]. The participators were equally categorized into three groups (A, B, and C) based on the amount of naswar consumed daily.

**Group A**. Mild naswar users have the habit of consuming up to half a packet of naswar per day (approx. <23g) for ≥12 months.
**Group B**. Moderate naswar users has the habit of consuming up to one packet of naswar per day (up to 45 g) for ≥12 months.
**Group C**. Severe naswar users have the habit of consuming one to two packets of naswar per day (45 to 90 g) for ≥12 months.

### Interview questionnaire

A preformed and structured questionnaire containing information regarding age, duration, frequency, and the number of packets of naswar consumed daily, site of placement of naswar intraorally, were asked and recorded. Moreover, tooth brushing frequency was also addressed and recorded for each participant. The questionnaire was prepared by three subject experts and validated by using Cronbach’s alpha (α) with coefficient of 0.80.

### Periodontal examination

The undermentioned periodontal clinical parameters were assessed on all teeth (except third molars) at six points (i.e. mesio facial, midfacial and disto facial, mesio lingual, mid lingual, and disto lingual), by a single calibrated examiner (AR), using UNC-15 probe and mouth mirror, and written down by trained recorders (ZA and MB).

- Bleeding on probing (BOP)–absence (0) or presence (1) of bleeding in 20–30 seconds after probing.
- Pocket depth (PD)–was evaluated to the closest mm at all six sites of each tooth (4-6mm and >6mm)
- Gingival Recession (GRec)–distance from the gingival margin to the CEJ (cementoenamel junction) to the closest achievable mm.
- Clinical Attachment Loss (CAL)–measured in mm from the cementoenamel junction to the bottom of the periodontal pocket.

### Statistical analysis

The collected data was analyzed with the help of IBM SPSS (statistical package for social sciences) Version 22.0. For all clinical parameters, descriptive statistics such as mean and standard deviation were calculated. To compare the means of the three groups, ANOVA was used. Statistical significance was deliberated at *p* value ≤0.05.

## Results

### Features of the study population

A total of 318 subjects were rounded up and examined for this study with 106 subjects in each (A, B, and C) group shown in Table 1. The subjects’ age varied from 17 to 44 years. The average age of the participants was 28.9±7 years. The patients who used naswar the most were 26, 27, and 28 years old, with 18 to 35 years old being the most common age group. Duration and continuance of naswar consumption, frequency of naswar placement, duration of naswar placement in the vestibule, and frequency of tooth brushing of the subjects are given in Table 2 The upper left quadrant was used by most naswar users (58.2%) for naswar dipping followed by upper right with 17%, lower left with 16.4%, and lower right with 8.5%.

**Table 1:** Groups of naswar users.

GROUPS	N	NO OF PACKETS (Naswar)	Weight
Mild Users	106	½ Packet	23g
Moderate Users	106	1 Packet	45g
Severe Users	106	1–2 Packets	45–90g

**Table 2:** Characteristics of naswar users.

CHARACTERISTICS	PERCENTAGE
**Duration of Naswar Consumption (in years)**	**%**
≤ 10	68
11–21	22
≥ 21	10
**Frequency of Naswar placement (Times per day)**	**%**
5–10	15
11–20	65
21–30	20
**Duration of Naswar Placement in vestibule (In minutes per day)**	**%**
5–10	20
11–20	65
21–30	15
**Frequency of Tooth Brushing**	**%**
Once a Day	68
Twice a Day	15
Once a week	2
Twice a week	5
3 times a week	10

### Periodontal clinical parameters

When comparing severe naswar users to moderate and light naswar users the periodontal parameters like bleeding upon probing, probing pocket depths (4-6mm and >6mm) gingival recession, and clinical attachment (mm) loss were considerably greater in severe naswar users with high statistical significance (p-value 0.001*). Light naswar users had to mean bleeding on probing percentage of 51.2%, and 56.6 and 61.9% in moderate and severe users respectively. Pocket depth (>6mm) mean percentage was highest in severe naswar users with 26.7%, followed by moderate users with 17.2% and light users with 12.5%. Severe naswar users had a high mean percentage of gingival recession (23.21%), while light users 13.6% and moderate users 14.8% had a smaller difference. Light naswar users showed 2.5% mean clinical attachment loss while in moderate and severe users it was 3% and 4.2% respectively (Table 3). Table 4 shows that ANOVA revealed a highly significant difference (p = .001*) among the three groups of naswar users. Bleeding on probing mean was 21.51 ± 1.66 (95% CI 21.20–21.84; p = 0.001) for mild Naswar users while that for severe users was 26.02 ± 0.71 (95% CI 25.88–26.16; p = 0.001). Similarly, mean of pocket depth (4-6mm) in mild users was 9.75 ± 0.837 (95% CI 9.59–9.92; p = 0.001) and mean of pocket depth (4-6mm) in severe users was 14.76 ± 0.834 (95% CI 14.60–14.92; p = 0.001).

**Table 3:** Mean percentages of clinical periodontal parameters.

Clinical parameters	Groups	N	Mean (%)	Lower Bound (95% CI)	Upper Bound (95% CI)
Bleeding on Probing	Mild	106	51.23	50.47	52
	Moderate	106	56.62	55.95	58.81
	Severe	106	61.95	61.62	62.28
Pocket depth 4-6mm	Mild	106	23.21	22.83	23.62
	Moderate	106	30.95	30.62	31.3
	Severe	106	35.14	34.76	35.52
Pocket depth >6mm	Mild	106	12.5	12.31	12.71
	Moderate	106	17.26	17.04	17.45
	Severe	106	26.78	26.38	27.16
Clinical attachment loss (in mm)	Mild	106	2.5	2.4	2.6
	Moderate	106	3	3	3
	Severe	106	4.25	4.17	4.34
Gingival recession	Mild	106	13.67	13.28	14.04
	Moderate	106	14.88	14.67	15.07
	Severe	106	23.21	22.83	23.57

**Table 4:** Comparison of clinical parameters among the three naswar groups.

Clinical Parameters	Groups	N	Mean	Std. Deviation	95% Confidence Interval for Mean		F value	P value
					Lower Bound	Upper Bound		
Bleeding on probing	Mild	106	21.52	1.669	21.20	21.84	291.2	0.001*
	Moderate	106	23.78	1.493	23.50	24.07		
	Severe	106	26.02	.717	25.88	26.16		
	Total	318	23.77	2.284	23.52	24.03		
Pocket depth 4-6mm	Mild	106	9.75	.837	9.59	9.92	1073.9	0.001*
	Moderate	106	13.00	.717	12.86	13.14		
	Severe	106	14.76	.834	14.60	14.92		
	Total	318	12.51	2.225	12.26	12.75		
Pocket depth >6mm	Mild	106	5.25	.438	5.17	5.34	2741.5	0.001*
	Moderate	106	7.25	.432	7.16	7.33		
	Severe	106	11.25	.837	11.08	11.41		
	Total	318	7.92	2.566	7.63	8.20		
Clinical attachment loss (in mm)	Mild	106	2.50	.502	2.40	2.60	585.2	0.001*
	Moderate	106	3.00	0.000	3.00	3.00		
	Severe	106	4.25	.438	4.17	4.34		
	Total	318	3.25	.833	3.16	3.34		
Gingival recession	Mild	106	5.74	.832	5.58	5.90	967.9	0.001
	Moderate	106	6.25	.432	6.16	6.33		
	Severe	106	9.75	.829	9.59	9.90		
	Total	318	7.24	1.925	7.03	7.45		

One-way ANOVA; statistical significance at p≤0.05;

* indicates high statistical significance.

**Fig 2:**
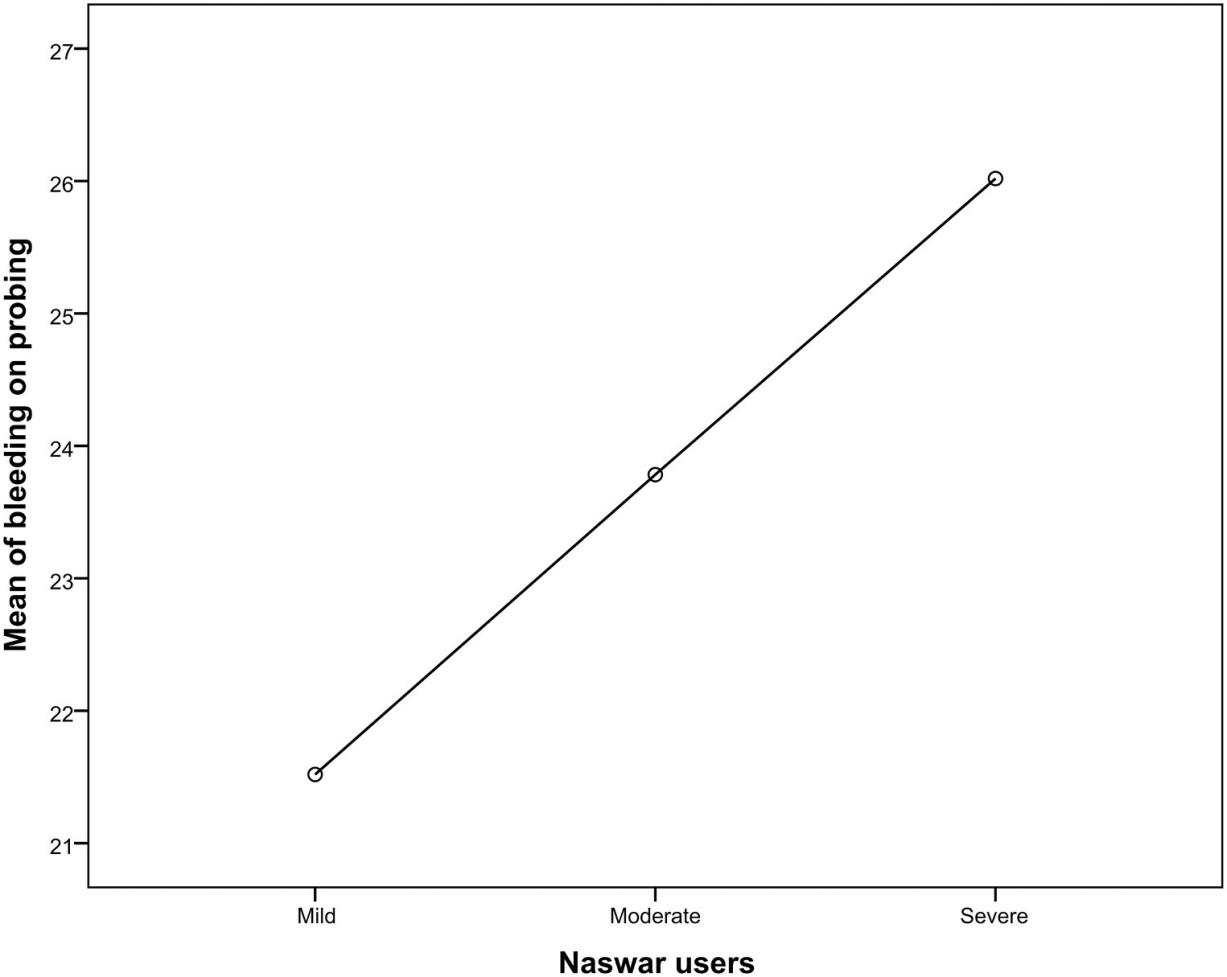
Mean score of bleeding on probing (BOP).

**Fig 3:**
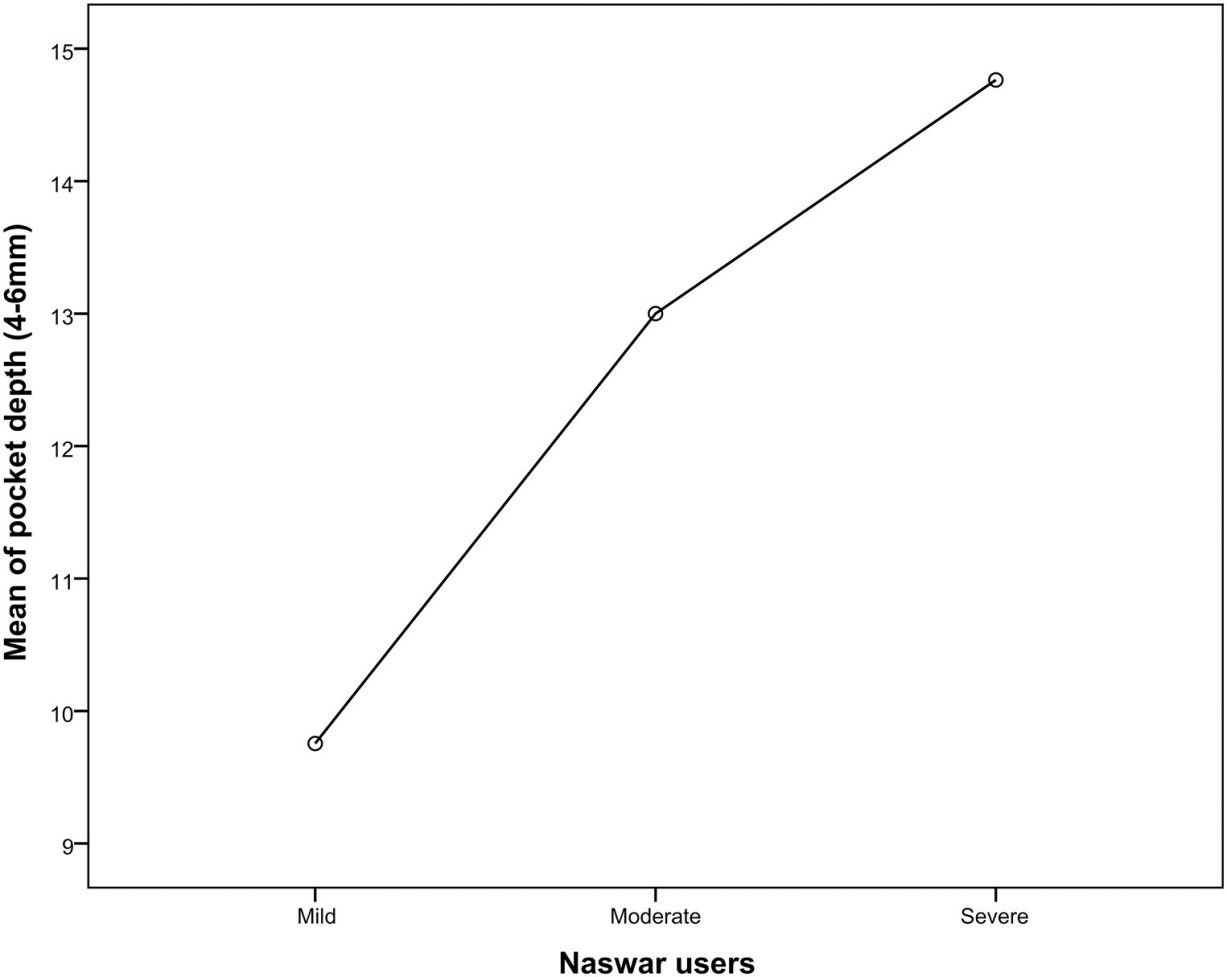
Mean score of pocket depth (4-6mm).

**Fig 4:**
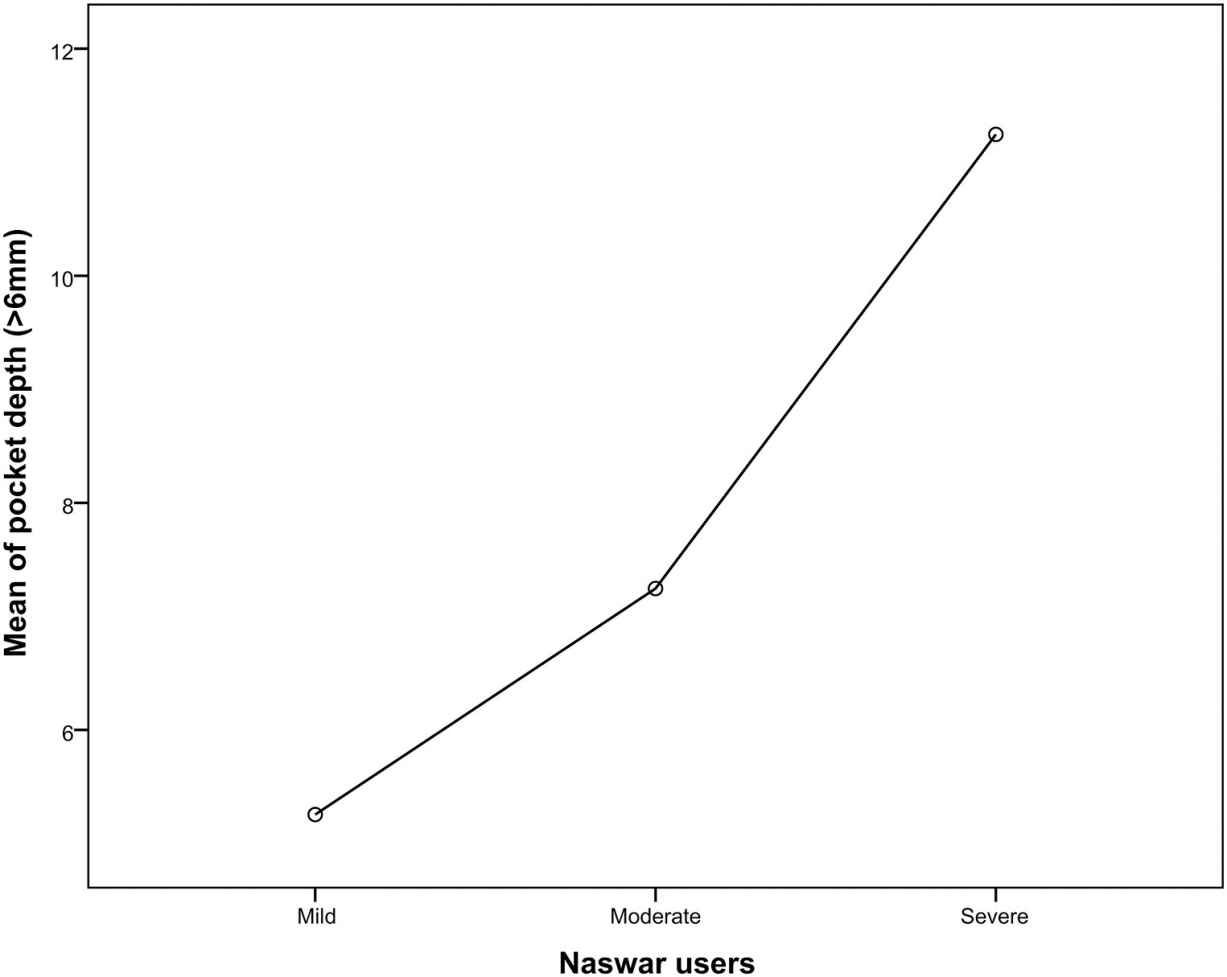
Mean score of pocket depth (>6mm).

**Fig 5:**
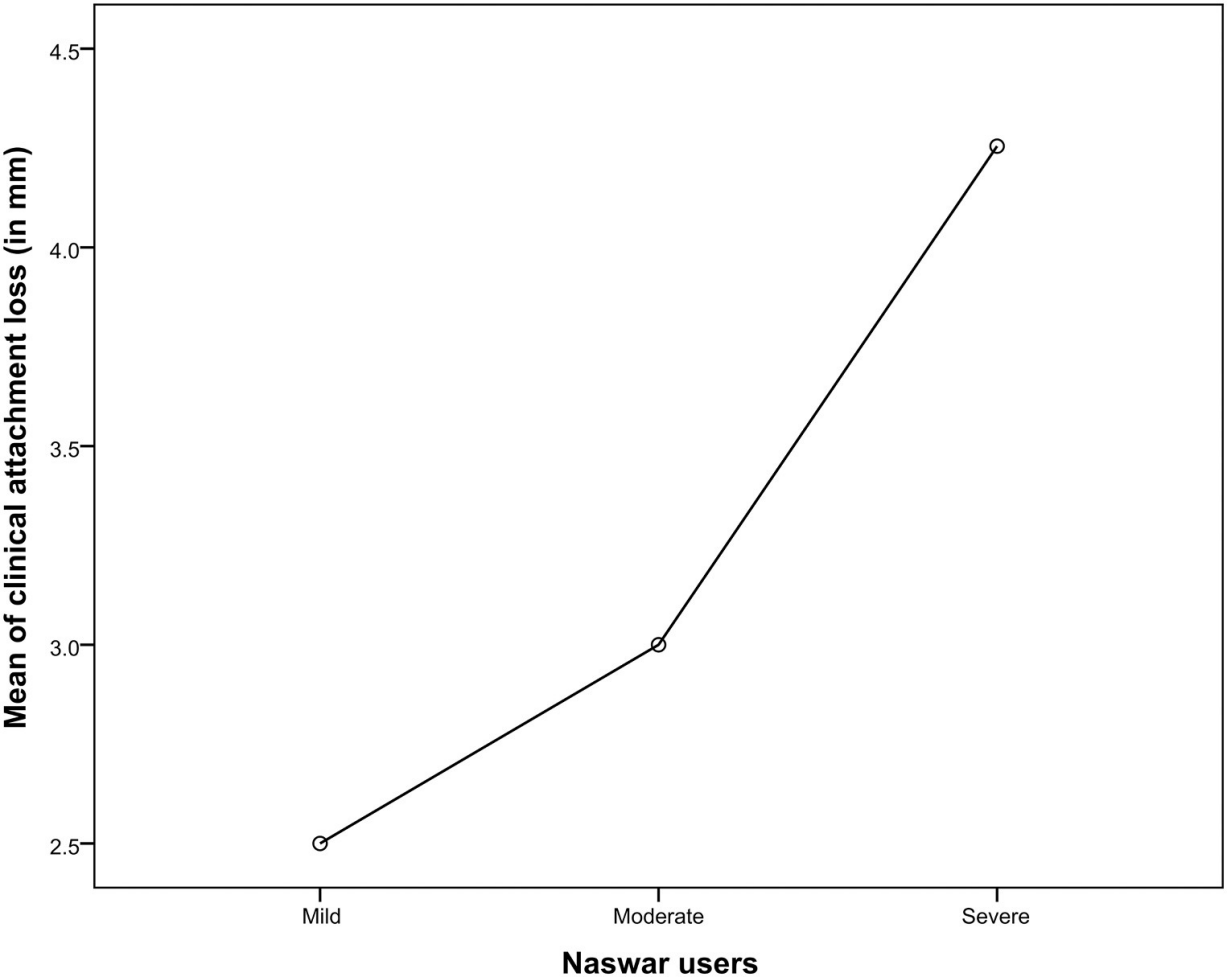
Mean score of clinical attachment loss, CAL (in mm).

**Fig 6:**
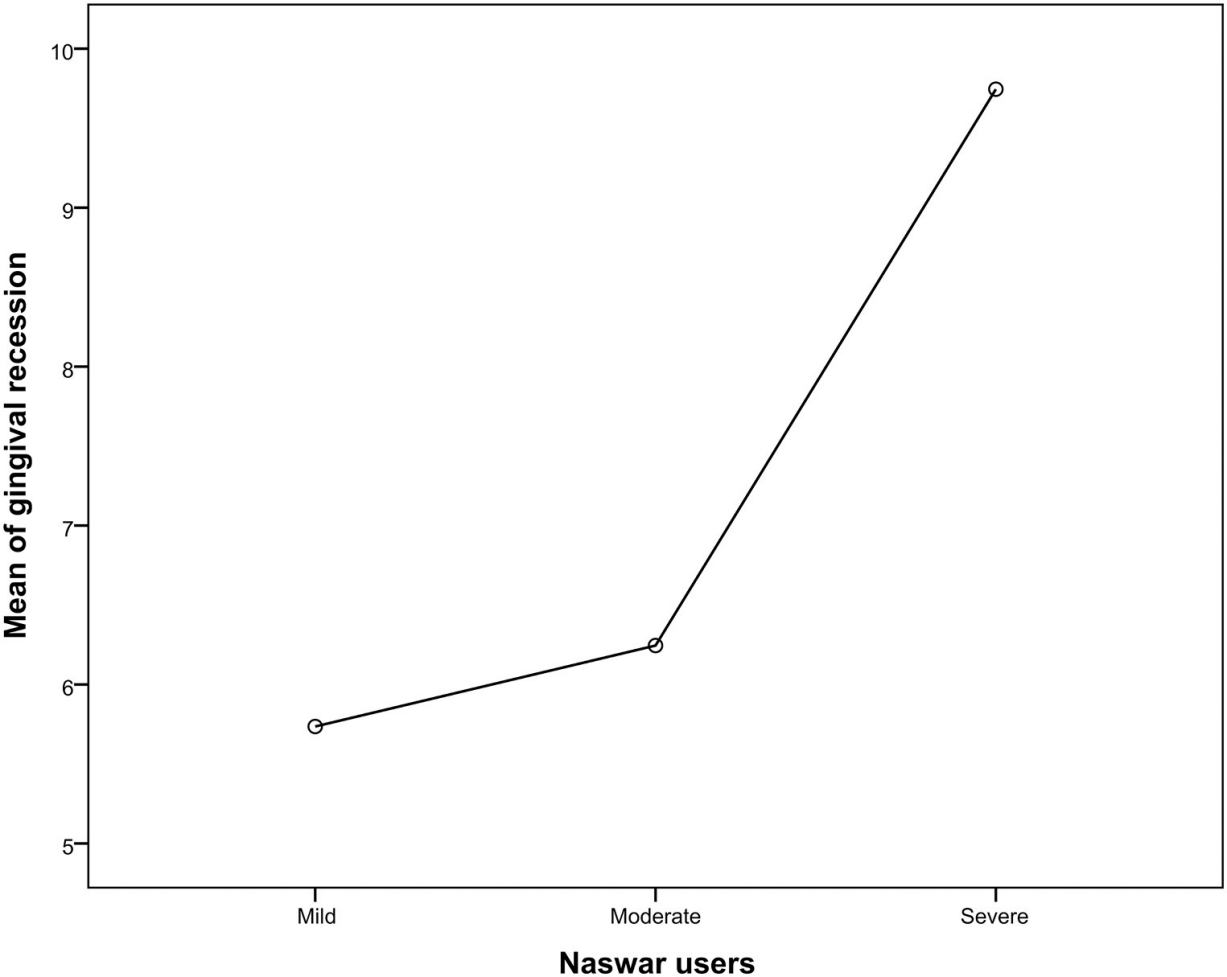
Mean score of gingival recession.

Relationship of various periodontal parameters against the naswar groups are shown in Figs 2–6.

## Discussion

Even though there have been several studies examining the influence of smokeless tobacco (naswar) usage on periodontal health, this is one of the first to record periodontal parameters in relation to the amount of naswar consumed (dose-response). This study assumed that the severity of the deterioration of periodontal clinical parameters worsens with an increase in the amount of naswar consumption. Results of this study bring out that bleeding upon probing, probing pocket depths, gingival recession, and clinical attachment loss significantly differ among the three groups of naswar (severe, moderate, and light users).

Tobacco is a momentous alterable hazard factor for poor periodontal health. Past research has shown that there is a significant link between tobacco use and several periodontal diseases [17,20–22]. This link was also discovered in studies involving periodontitis therapy. Tobacco consumption has been found to have a negative impact on probing pocket depth after periodontal therapy and long-term prognosis by previous researchers [23]. These studies continue to be relevant to this research. According to Al-Askar et al and Javed F. et al, patients who use smokeless tobacco product/s have the poorest periodontal parameters in comparison to those who do not use such product/s [4,16]. This might be because of raise in the level of reactive oxygen species and pro-inflammatory cytokines like IL-1b, IL-6, TNF-α, and MMP-8,9 due to pulverized tobacco in tobacco users [24]. Furthermore, nicotine in tobacco activates the sensory nerves causing the release of vasodilatory peptides from the peripheral terminals resulting in hyperaemia in the gingival blood vessels [25]. Besides, ex vivo studies have shown that nicotine affects gingival fibroblasts proliferation and function, enhances expression of integrin β-1, decreases the production of collagen, increases collagenase activity of fibroblasts, and loss of alveolar bone [12,26–29]. These might be contributing causes to considerably greater percentages of bleeding on probing, periodontal depths (4–6 & >6), gingival recession, and clinical attachment loss in mm.

The duration and frequency of use of tobacco product/s are linked to the severeness of oral inflammatory conditions, inclusive of periodontal diseases and oral malignant change [30,31]. According to Sing et al, those who have had smokeless tobacco products for more than five years had severe inflammation of the periodontium than people who have used them for less than five years [30]. This research is in line with this study. Nonetheless, the findings of Akram et al showed no statistically significant difference in the grievousness of periodontal parameters of five years and ten years users of naswar and gutka. They deduced that people who have used smokeless tobacco for a shorter time of up to five years are as susceptible to periodontal diseases as people who have used it for quite a long period of more than ten years, which contradicts this study, which found that people who used naswar for a long time had higher scored of periodontal parameters [4].

Daood et al found that the radiographic and clinical periodontal parameters were more regretful in the naswar group than in the control group, in another investigation. The use of naswar resulted in a greater amount of breakdown of collagen in the connective tissue of the naswar group, which validates the findings of this study [15]. A study by Al-Hamoudi et all revealed a substantial difference between naswar users and non-users. It disclosed that naswar users had the worst clinical and radiographic periodontal parameters along with compromised self-perceived oral signs and symptoms [32]. It was also proposed by them that dipping naswar should be considered a possible danger for periodontium. This study’s findings corroborate the findings of this study.

The most common age group that participated in this study was 18 to 35 years old, with a higher number of people in their 30s. The upper left quadrant was similarly shown to be the most common site for naswar dipping (58.2%) in this investigation. These are the distinguishing features of this study.

## Conclusion

In inferred deduction, periodontal clinical parameters were worse in heavy naswar users followed by moderate and mild naswar users, which suggests that an increase in consumption of naswar quantity increases the severity of the deterioration of periodontal clinical parameters.

## Supporting information

S1 Dataset (SAV)

S2 Dataset (SAV)
